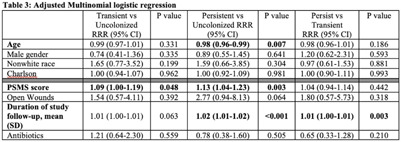# Low function and frequent readmission in nursing home patients with persistent resistant gram-negative bacterial colonization

**DOI:** 10.1017/ash.2022.158

**Published:** 2022-05-16

**Authors:** John Mills, Julia Mantey, Marco Cassone, Keith Kaye, Lona Mody

## Abstract

**Background:** Persistent colonization with resistant gram-negative bacteria (R-GNB) increases risk of clinical infection and intra-facility transmission among nursing home (NH) patients. Limited data exist on the roles of age and function on duration of R-GNB colonization. **Methods:** Secondary data analysis was performed from a cohort study of patients admitted to 6 Michigan NHs between November 2013 and May 2018. Swabs obtained upon enrollment, day 14, day 30, then monthly until NH discharge from 6 anatomical sites were cultured for GNB. R-GNB were defined as resistant to ciprofloxacin, ceftazidime, or imipenem. Positive R-GNB culture from a single visit followed by negative cultures for the same organism from ≥2 subsequent visits were defined as transient R-GNB colonization. All other patients with positive R-GNB cultures from multiple visits were considered persistently colonized. Demographic data, antibiotic use, device use, and physical self-maintenance scales (PSMSs) were obtained upon enrollment. Characteristics were compared between patients with transient versus persistent R-GNB and uncolonized patients using multinomial logistic regression. **Results:** We recruited 896 patients (median age, 75 years; 41% male; 46% nonwhite) and followed them for 2,437 total visits. Of 896 patients, 407 (45.4%) were colonized with ≥1 R-GNB during their stay. Of 171 patients with ≥ 2 follow-up visits after R-GNB detection, 94 (55%) remained persistently colonized with the same R-GNB (Table 1). *Escherichia coli* (30%) and *Proteus mirabilis* (22%) were the most frequently identified R-GNB. The most common anatomical colonization sites were perirectal (368 [24.3%] of 1,147) groin (340 [14.3%] of 2,046), and hands (115 [4.8%] of 2283). Compared to uncolonized patients, patients with persistent (1.09; 95% CI, 1.00–1.19, *P* = .048) and transient R-GNB colonization (1.13; 95% CI, 1.04–1.23; *P* = .003) had lower PSMS (Tables 2 and 3). Compared to uncolonized and transiently colonized patients, patients with persistent R-GNB colonization had prolonged lengths of NH stay (1.01; 95% CI, 1.00–1.01; *P* = .003). **Conclusions:** R-GNB colonization in vulnerable NH patients is common (407 [45.5%] of 896 and often persistent (94 [55%] of 171 patients with sufficient follow-up to assess persistence). Patients with persistent R-GNB had lower functional status, longer LOS, and higher readmission rates than those without. R-GNB decolonization should be investigated as a strategy to potentially improve outcomes among NH patients.

**Funding:** None

**Disclosures:** None

**Table:**
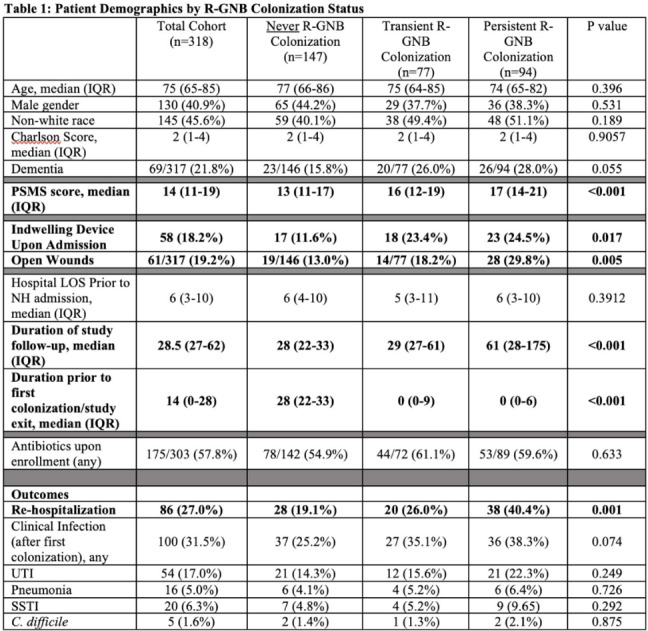

**Table:**
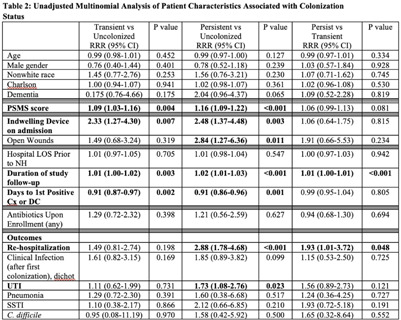

**Table:**